# The Digestive Tract of Cephalopods: a Neglected Topic of Relevance to Animal Welfare in the Laboratory and Aquaculture

**DOI:** 10.3389/fphys.2017.00492

**Published:** 2017-07-17

**Authors:** António V. Sykes, Eduardo Almansa, Gavan M. Cooke, Giovanna Ponte, Paul L. R. Andrews

**Affiliations:** ^1^Centro de Ciências do Mar do Algarve, Universidade do Algarve Faro, Portugal; ^2^Centro Oceanográfico de Canarias, Instituto Español de Oceanografía Santa Cruz de Tenerife, Spain; ^3^Department of Life Sciences, Anglia Ruskin University Cambridge, United Kingdom; ^4^Association for Cephalopod Research (CephRes) Naples, Italy; ^5^Department of Biology and Evolution of Marine Organisms, Stazione Zoologica Anton Dohrn Villa Comunale, Naples, Italy

**Keywords:** cephalopods, digestive tract, Directive 2010/63/EU, *Octopus vulgaris*, *Sepia officinalis*, welfare

## Abstract

Maintenance of health and welfare of a cephalopod is essential whether it is in a research, aquaculture or public display. The inclusion of cephalopods in the European Union legislation (Directive 2010/63/EU) regulating the use of animals for scientific purposes has prompted detailed consideration and review of all aspects of the care and welfare of cephalopods in the laboratory but the information generated will be of utility in other settings. We overview a wide range of topics of relevance to cephalopod digestive tract physiology and their relationship to the health and welfare of these animals. Major topics reviewed include: (i) Feeding cephalopods in captivity which deals with live food and prepared diets, feeding frequency (*ad libitum* vs. intermittent) and the amount of food provided; (ii) The particular challenges in feeding hatchlings and paralarvae, as feeding and survival of paralarvae remain major bottlenecks for aquaculture e.g., *Octopus vulgaris*; (iii) Digestive tract parasites and ingested toxins are discussed not only from the perspective of the impact on digestive function and welfare but also as potential confounding factors in research studies; (iv) Food deprivation is sometimes necessary (e.g., prior to anesthesia and surgery, to investigate metabolic control) but what is the impact on a cephalopod, how can it be assessed and how does the duration relate to regulatory threshold and severity assessment? Reduced food intake is also reviewed in the context of setting humane end-points in experimental procedures; (v) A range of experimental procedures are reviewed for their potential impact on digestive tract function and welfare including anesthesia and surgery, pain and stress, drug administration and induced developmental abnormalities. The review concludes by making some specific recommendations regarding reporting of feeding data and identifies a number of areas for further investigation. The answer to many of the questions raised here will rely on studies of the physiology of the digestive tract.

## Introduction

Normal development, growth and the maintenance of health and well-being are only possible if all the digestive tract functions (e.g., motility, digestion, and absorption) operate normally and in concert. Understanding the physiological processes and the impact of external factors (e.g., handling, temperature, diet quality including exposure to food toxins, exposure to viral/bacterial infections and parasites) is important for normal laboratory maintenance of the animal in a research setting, as well as for optimizing conditions for aquaculture at each life stage.

The study of the physiology of the cephalopod digestive apparatus has mainly focused on *Sepia officinalis* (Bidder, 1966; Boucaud-Camou and Boucher-Rodoni, 1983; Mangold and Bidder, 1989; Quintela and Andrade, 2002a,b; Sykes et al., 2013; Costa et al., 2014), *Octopus vulgaris* (Boucher-Rodoni and Mangold, 1977; Boucaud-Camou and Boucher-Rodoni, 1983; Andrews and Tansey, 1983b; Mangold and Bidder, 1989), *Octopus maya* (Martínez et al., 2011a,b, 2012; Rosas et al., 2013; Linares et al., 2015; Pech-Puch et al., 2016). Few studies have been carried out in *Loligo vulgaris* and other squid (Bidder, 1950; Mangold and Bidder, 1989). Furthermore, the morphology, motility and absorptive functions of the digestive tract of *Nautilus pompilius* have been the subject of limited investigation (Westermann and Schipp, 1998a,b, 1999; Ruth et al., 1999; Westermann et al., 2000, 2002).

The inclusion of all “live cephalopods,” taken to mean all living species (about 700), at all life stages after hatching, in Directive 2010/63/EU (European Parliament and Council of the European Union, 2010) covering the use of animals in scientific research and education poses a number of challenges for research (Smith et al., 2013; Fiorito et al., 2015) including that aimed at optimizing practices in aquaculture (Sykes et al., 2012; Smith et al., 2013; Fiorito et al., 2015). Whilst the Directive regulates studies in the Member States of the European Union, the principles it enshrines and the approaches to care and welfare required for compliance are likely to impact on cephalopod research outside the European Union (see Fiorito et al., 2014, for discussion of wider implications). In comparison to the commonly studied vertebrate laboratory species and commercially exploited vertebrates such as salmon and trout, chickens, cows, and pigs (Stevens, 1988; Grosell et al., 2010; Rønnestad et al., 2013), knowledge of the physiology of the cephalopod digestive tract at all life stages is limited.

Cephalopods are also kept for education and display purposes and, as in the laboratory and aquaculture, the normal functioning of the digestive tract is essential for good health and wellbeing (Fiorito et al., 2015). In this review, we will highlight a number of specific aspects of the relationship between feeding behavior and the physiology of the cephalopod digestive tract where increased understanding is required to ensure animal welfare. We will also discuss areas where further study is required.

## Feeding cephalopods in captivity

The vast majority of known cephalopods are carnivorous, obtaining food either by scavenging (e.g., *N. pompilius*) or predation of live food (e.g., *S. officinalis, L. vulgaris, O. vulgaris*), with a few exceptions such as the vampire squid (*Vampyroteuthis infernalis*) that is a detritivore of “marine snow,” possibly requiring different digestive processes to obligate cephalopod carnivores (Hoving and Robison, 2012).

Information on diets in the wild is limited, but insights can be obtained through analysis of isotopes or stomach contents among other techniques (e.g., Hobson and Cherel, 2006; Villegas et al., 2014; Scheel et al., 2016; Regueira et al., 2017). In captivity, individual animals may show a preference for one type of prey over another even when both are available (e.g. crustaceans vs. fish for *S. officinalis*, crustaceans vs. mussels for *O. vulgaris*). Captive conditions, especially during key developmental periods may influence food preference across the life of an individual, as seen for example in cuttlefish (Darmaillacq et al., 2008). Nevertheless, most of the species adapt well to the laboratory and the diet may be narrowed to a few selected items (see Fiorito et al., 2015).

### Live food and prepared diets

The provision of live food (mostly commonly crustaceans, mussels, or small fish) to captive cephalopods is the most common practice. It is normally based on freshly caught (when applicable) items, that are not subjected to nutritional decay (e.g., oxidation in frozen food) as it may affect the growth of the cephalopod (Correia et al., 2008a,b). Live food greatly increases husbandry requirements as most cephalopods require crustaceans which produce higher levels of waste material (i.e., exoskeleton material containing uneaten soft tissues), thus potentially affecting water quality in tanks; this puts an additional burden on filtration systems and cleaning logistics. Providing live food also raises ethical issues as the death of the prey may not be humane, especially if it has the *potential* to suffer as fish are assumed to (Sneddon, 2015), and decapod crustaceans (Barr et al., 2008) may do.

Cephalopods are likely to have been exposed to parasites or toxins from their diet in the wild, however concern in the use of live food for laboratory housed animals may be also taken into account because of the hypothetical risk posed by introduction through the food given.

Contrarily, the use of live food is simply neither practical nor economical in production scale aquaculture so there is a considerable research effort into the development of prepared diets (Iglesias et al., 2014; Martínez et al., 2014; Cerezo Valverde and García, 2017). If successful, these alternative feeds would also possibly become a standard in the laboratory and public aquaria.

Although prepared diets may be cheaper, are likely to be more convenient and have a lower potential infection risk than live food, there is an argument that the provision (possibly intermittently) of live or frozen prey as food can be considered as “enrichment” for the cephalopod and hence beneficial for overall welfare (Cooke and Tonkins, 2015; see review in Fiorito et al., 2015). The importance of environmental (“tank”) enrichment in animal experimentation (Bayne and Würbel, 2014; Yasumuro and Ikeda, 2016) and aquaculture (Ashley, 2007; Martins et al., 2012; Näslund and Johnsson, 2016) is being increasingly appreciated.

### Feeding frequency and the amount of food provided

In the wild, the frequency of food intake in a cephalopod is considered to be influenced by multiple factors including: the availability of prey; the ability of the animal to locate and catch the prey (this will be affected by its health status); the time taken to ingest the prey and any pre-absorptive satiety signals (e.g., crop or stomach distension via mechanosensitive afferents if they exist in cephalopods); the time taken to digest the prey, absorb the nutrients and eliminate waste (all likely to be affected by any digestive tract pathology; see below) together with any post-absorptive satiety signals; the size, amount and nutritional value of the prey; the metabolic rate of the animal; activity level; environmental temperature and seasonality; life stage and sexual maturity.

Food availability and seawater temperature are considered to be the major factors influencing growth of cephalopods (Semmens et al., 2004). However, even if these parameters are optimal with respect to animal needs, if the digestive tract is in a pathophysiological state, food utilization will be affected with negative impact on bodily maintenance, growth and overall health.

In the laboratory or other captive settings, feeding frequency and amount of food provided may also considered within the framework of the “Five Freedoms” principle outlining minimal welfare standards (http://ec.europa.eu/food/animals/welfare_en; Huntingford, 2008). One of the “Freedoms” states that animals should be “free from hunger” (www.rspca.co.uk/education). It is not known if cephalopods experience a sensation of hunger, or a functionally equivalent sensation, comparable to that in humans. Tracking the behavioral changes such as latency and time to attack, following a large meal (“satiety”) to the time when the meal has been fully digested (presumed presence of “hunger”) will provide insights into the frequency with which food should be provided to minimize the possibility of unpleasant sensations arising from lack of food (i.e., hunger). Below we will focus on two modalities of providing food to animals: *ad libitum* and intermittent.

*Ad libitum* feeding is usually taken to mean that the animal has access to an “excess” amount of food at all times so that in principle it can eat to satiety (or potentially over feed) whenever it requires. However, few studies define what they mean by *ad libitum*. Even when the frequency of food provision is stated the amount provided is not, making comparison of regimes difficult. For example, an *ad libitum* regime in juvenile *S. officinalis* is provided in some settings by live grass shrimp (*Palaemonetes varians*) given twice daily although the amount given is not specified (Gonçalves et al., 2012). Examples of species in which feeding described as *ad libitum* feeding has been used include: for sepiolid, *Euprymna tasmanica*, (live mysids, Moltschaniwskyj and Johnston, 2006); for cuttlefish, *S. officinalis*, (live and frozen grass shrimp, Sykes et al., 2013); for octopus, *O. vulgaris*, live crabs (e.g., Agnisola et al., 1996); for *O. maya* (frozen crabs, Linares et al., 2015). *Ad libitum* feeding is achieved by placing food in the tank to allow the animals free access, with sufficient food provided to last until replenished.

An alternative may be to re-examine the utility of automated food dispensers previously investigated for example for feeding *O. vulgaris* (Nixon, 1969). Such automated methods could easily be applied to pelleted diets (artificial or synthetic) and would allow the feeding pattern to be studied which will be essential for investigating the neural and hormonal mechanisms controlling food intake and digestion. With a food dispenser there is a possibility that the animal will access, but not eat food, increasing wastage and tank contamination. Additionally, animals housed in groups (e.g., *O. vulgaris* in some aquaculture settings) may compete for access to the dispenser. There is also a possibility that the animal (particularly octopuses) will damage the food dispenser or may damage themselves on the food dispenser.

For aquaculture, *ad libitum* feeding will be necessary to ensure fastest growth rates although the health and welfare consequences (if any) of such regimes require study. However, it is reported that it is impossible to over feed a cephalopod and that they will reject excess food, but the evidential basis for this appears unclear (Boucaud-Camou and Boucher-Rodoni, 1983) although it is consistent with the experience of one of the authors of this review (AS).

*Intermittent feeding* applies to conditions where a given amount of food is provided to the animal which it will consume in one “meal” and it is most commonly applied to providing food either daily (e.g., grass shrimp for *Abdopus aculeatus*, Alupay et al., 2014; pieces of fish for *O. vulgaris*, Matzner et al., 2000; García García et al., 2011), more frequently (e.g., frozen squid twice daily for *O. vulgaris*, Garcia-Garrido et al., 2010) or less frequently (e.g., a crab every second day in *O. vulgaris*, Boucher-Rodoni and Mangold, 1985; shore crab twice weekly in *S. officinalis*, Wearmouth et al., 2013). Food is not available at all times but obviously if the amount of food provided is greater than can be ingested in a single meal then in effect food is provided *ad libitum*. For example, in *Octopus maya* animals given one small crab/50 g body weight daily ate all the crab, but if two crabs/50 g body weight were provided at the same time animals did not ingest all the crab (Walker et al., 1970). The literature also has examples of feeding an individual “meal,” but providing food on multiple occasions during the day. For example, Yacob et al. (2011) report feeding *S. officinalis* on frozen shrimp and live zebra fish twice daily and in another cuttlefish study animals were fed frozen krill and silversides by hand 3–4 times a day (Tressler et al., 2014).

A further example of issues relating to the description of feeding regimes is provided by a study of *O. vulgaris* which comments, “octopuses were fed once per day to satiation with filet of frozen bogue (*Boops boops*)” (García García et al., 2011, p. 162). It is presumed that satiety was indicated by the refusal to take further food when offered but it would not necessarily be known if the animals would eat again if offered food prior to the next day. If the term “satiety” is used it would be helpful to have an indication of the criteria on which the assessment was made.

It is evident that the Methods sections of publications do not always give sufficient information either for the feeding regime to be replicated or to enable valid inter-study comparison. In view of this we would recommend that as a minimum the Authors should specify: the type of food provided (e.g., live, freshly killed, previously frozen, processed/prepared); the amount provided (weight, including for live food; see Methods in Lamarre et al., 2012, for an example); the frequency of feeding and the time at which the food was provided.

For example, Garcia-Garrido et al. (2010) report feeding *O. vulgaris* on frozen squid (*Loligo gahi*) given at two times (9 a.m. and 3 p.m.), with the total amount of food provided daily being 5% of the body weight of the octopus. Ideally the energy density and chemical composition of the diet should also be given (for an example see Estefanell et al., 2011) or a reference provided to previous studies where similar food has been provided.

### Food amount

Despite the diverse feeding regimes and paucity of detail in much of the literature, a number of publications have undertaken detailed studies of the feeding requirements of several species (e.g., *O. vulgaris*, Estefanell et al., 2011; *Octopus tetricus*, Joll, 1977; *S. officinalis*, Domingues et al., 2001). Feeding requirements of a particular species, at a particular life stage and temperature are usually given as the weight of food as a percentage of body weight. The latter assumes the energy density of the food is sufficient to meet all the calorie and nutrient requirements and micronutrients. However, not all diets are equivalent. For example, in adult *O. vulgaris* growth rate is lower when they are fed *ad libitum* exclusively on sardines compared with squid or crab (Quintana et al., 2015). One reason to explain this difference could be the high neutral lipid content of sardines. Cephalopods seem to have a low capability to digest neutral lipids due to the absence of lipid emulsifiers in their digestive tracts (Vonk, 1962; O'dor et al., 1984; Morillo-Velarde et al., 2015).

For a particular diet, under well-defined environmental conditions (particularly temperature) it should be possible to give guidance on the amount of food required daily for a given species and life stage. This approach is illustrated by the study of Garcia-Garrido et al. (2010) in which *O. vulgaris* juveniles were fed 5% of their body weight daily on a diet of frozen squid or by the studies of Castro et al. (1993), Castro and Lee (1994), and Domingues et al. (2008), where *S. officinalis* were fed ≈8% of their body weight daily on shrimp species (either *Palaemon* or *Palaemonetes*).

As publications adopt a more systematic and detailed approach to describing diets in the methods sections it will be possible to undertake meta-analyses of the dietary data and provide stronger evidence based guidance on the amount of a particular food to provide.

Studies of feeding regimes are currently driven mainly by the aquaculture potential of cephalopods and the requirement to either develop artificial diets (Iglesias et al., 2014; Vidal et al., 2014) or to optimize natural diet formulations (e.g., ratio of fish to crab for *O. vulgaris*; García-García and Cerezo-Valverde, 2006). It is essential that the diet provided in the laboratory, aquaculture or public aquaria fulfills all the nutritional requirements of the animal, including micronutrients (Navarro et al., 2014). In this context, studies comparing metabolism of animals fed natural and artificial diets are of particular relevance (e.g., *O. maya*, Rosas et al., 2007). Studies of food intake and growth can be supplemented by measurements of haemolymph protein, amino acids and long chain polyunsaturated fatty acids (Linares et al., 2015), digestive enzymes (e.g., Villanueva et al., 2002) or body composition to provide an objective assessment of metabolic status (Garcia-Garrido et al., 2010; Navarro et al., 2014; Linares et al., 2015).

### Particular challenges in feeding hatchlings and paralarvae

Cephalopods do not undergo metamorphosis like fish larvae (Young and Harman, 1988) but several species have a first life stage that starts with hatching and lasts until all its systems mature, including the digestive tract. Nonetheless, as in fish (Zambonino-Infante and Cahu, 2001), this period of maturation is normally a period of a month, depending on seawater temperature (Moguel et al., 2010; Sykes et al., 2013; Iglesias and Fuentes, 2014), and has been identified as a recurrent bottleneck for the development of cephalopod aquaculture (Sykes et al., 2006; Villanueva et al., 2014). Despite the multitude of reproductive strategies that cephalopods display (Rocha et al., 2001), the first life stage after hatching is usually termed “paralarvae” for species that undergo indirect embryonic development, and “hatchlings” for those which display direct development. These differences in development correspond to differences in the number and size of eggs laid by females (higher in indirect development species, e.g., *O. vulgaris*) and the relative “degree” of development at hatching (higher in direct development species, e.g., *S. officinalis, O. maya*; Boletzky, 1981, 1986). This is the key point leading to particular challenges regarding welfare of paralarvae and hatchlings in both the laboratory and aquaculture. In both cases (either paralarvae and hatchlings), cephalopods kept under captivity hatch with inner yolk reserves (Boletzky, 1994) that may last for days or weeks depending on temperature and proper food availability, as they display a mixed embryonic and postembryonic nutrition (Boletzky and Villanueva, 2014). Despite the differences in size at hatching, neither paralarvae nor the hatchlings display a prey ingestion problem related to the size of the “mouth,” as that reported for finfish (Yúfera and Darias, 2007). However, the use of any feeds, other than live prey, during this first life stage (Sykes et al., 2014) limits growth and development, and eventually will impact welfare, (Navarro and Villanueva, 2000; Sykes et al., 2013).

## Parasites and toxin load of the cephalopod digestive tract: possible impact on animal welfare and confounding variables in experimental design

The wild remains the main source for cephalopods used in research so there is a realistic possibility that animals in the laboratory may have parasites in the digestive tract. Parasites are of concern for several reasons including, the impact on the overall health and welfare of the animal (Sykes and Gestal, 2014), as a source of infection for other animals (including humans unless precautions are taken, see Fiorito et al., 2015), and as a confounding factor in an experiment.

The digestive tract parasite *Aggregata octopiana* (Protozoa: Apicomplexa) is considered to be one of the main epizootic agent in both wild and reared *O. vulgaris* and exemplifies some of the issues. The intermediate host for this coccidian are crustaceans that form part of the normal diet for *O. vulgaris. Aggregata* is reported to cause loss of epithelial cells, mucosal atrophy and inflammation in the caecum and intestine resulting in impaired body growth, proposed to all be due to intestinal malabsorption syndrome (Gestal et al., 2002a,b). The prevalence of *A. octopiana* is high and reported to reach 98% (Gestal et al., 2002a) in the local population (Vigo, Spain). Although it is possible to count the number of sporocysts to assess the presence and magnitude of infection (Castellanos-Martinez et al., 2014) this requires death of the animal and removal of gut tissue, so it is not currently possible to assess the presence of *Aggregata* at the time animals are allocated to an experimental group. Whilst random allocation should reduce the potential for inadvertently assigning animals with high and low levels of infection to “control” and “test” groups for an experiment, it is possible that including groups with some infected animals may increase the variability within the group, potentially leading to a false negative result from statistical testing. In addition, the variability will affect the validity of any power calculation used to estimate group size in experimental design if the estimated effect size is influenced by the parasitic infection.

In theory, it should be possible to identify animals infected with *Aggregata* by looking for sporocysts in the feces of living animals prior to allocation to study groups, but this possibility has not been explored in published accounts, to the best of our knowledge. In *O. vulgaris*, it may also be possible to inspect the terminal intestine for sporocysts by gentle retraction of the ventral mantle or by endoscopy. Until techniques are available for *in vivo* assessment of *Aggregata* status or for its eradication prior to experiment it is probably wise to assume a high prevalence of infection in animals caught from the wild, unless there is evidence to the contrary in a local population used by a particular laboratory. We recommend that as a minimum the digestive tract is carefully examined macroscopically *post mortem* for the presence of sporocysts, particularly in the caecum and intestine but they may also be visible in the crop. Ideally the number of *Aggegata* sporocysts should be counted so that the magnitude of any infection can be investigated for correlation with experimental parameters to build up a profile of its biological effects. Once the spectrum of effects of *Aggregata* infection is known it will then be possible to make an informed assessment about its role as a confounding factor in experiments.

Whilst we have focused on *Aggregata* as a potential confounding factor in research we should not overlook the fact that animals with any infection may have a lower health status than those without and this raises a broader question of whether animals with *Aggregata* should ever be used in research studies other than those studying the effects of *Aggregata* itself.

We have used the infection of the digestive tract by *Aggregata* to illustrate one potential problem with using wild caught animals but we should also consider the digestive tract flora. The digestive tract microbiome and its role in health and disease has been the subject of detailed investigation in mammals but studies of the digestive tract flora are required in cephalopods. We mention the digestive tract microbiome here in relation to welfare as when animals are transferred from the wild to the laboratory, possibly involving a change of diet, it is highly likely that the flora in the digestive tract will change with as yet unknown effects on the functionality of the digestive tract and overall animal health. In addition, the impact of feeding prepared diets on the digestive tract flora of either wild caught or laboratory bred animals is not known.

A final factor to consider is the impact of any toxins that the animal may have been exposed to in the wild and which gain access to the body via the digestive tract. To illustrate this point we will use as examples shellfish toxins produced by phytoplankton species, ingested by bivalves which are subsequently ingested by benthic cephalopods. Domoic acid is responsible for amnesic shellfish poisoning in mammals (Pulido, 2008; Lefebvre and Robertson, 2010) and several compounds (e.g., saxitoxin, neosaxitoxin, gonyautoxins) are responsible for paralytic shellfish poisoning (Lopes et al., 2013). In mammals, these shellfish toxins have acute onset (<24 h) neurological (e.g., amnesia and locomotor paralysis) and gastrointestinal (nausea, vomiting and diarrhea) effects (for reviews see Lefebvre and Robertson, 2010; Visciano et al., 2016).

In cephalopods, although there are reports of mass stranding of Humboldt squid (*Dosidicus gigas*; Lopes et al., 2013) attributed to paralytic shellfish toxins (PST), we were unable to find any reports of acute onset effects of PSTs. In a study of PSTs in *O. vulgaris*, Lopes et al. (2014) comment (p. 210), “Despite of the remarkably high levels of toxins detected no apparent harm neither signs of behavioral changes were observed.”

For the amnesic shellfish toxin domoic acid reports of acute effects are also lacking, an observation that is particularly intriguing as glutamate is known to be a neurotransmitter in both central and peripheral neural tissues in cephalopods (Messenger, 1996). Both domoic acid and PSTs have been shown to accumulate in the digestive gland of *O. vulgaris* (Costa and Pereira, 2010; Lopes et al., 2014) but the effects of these toxins on digestive gland function or other tissues is not known. Animals caught from the wild and utilized in research studies are likely to have different digestive gland concentrations of amnesic and paralytic shellfish toxins and these may vary seasonally depending upon algal blooms. The impact of the “toxin load” on health of the animal is unknown and differences in toxin concentration are a possible contributor to experimental variability; research is required to characterize the acute (e.g., brain and digestive tract function) and chronic (e.g., digestive gland metabolism, animal growth, and ability to withstand infection) physiological effects of amnesic and paralytic shellfish toxins on the cephalopods commonly studied in the laboratory.

## Food deprivation as a component of research investigations

There are several situations in research when it may be considered necessary to deliberately deprive the animal of food. Here we consider the justification for food deprivation in different contexts and also the duration of such deprivation within the framework of regulated procedures under Directive 2010/63/EU (European Parliament and Council of the European Union, 2010).

### Transportation

Accumulation of toxic ammonia from renal excretion arising primarily from protein metabolism (García García et al., 2011) and fecal contamination are a risk when transporting cephalopods in non-circulating sea water resulting in acidification. Depending upon the transport distance, species and size of the animal food deprivation should be considered and may be combined with transport in water at a temperature below ambient to reduce metabolic rate and carbon dioxide production (Fiorito et al., 2015).

There are no specific recommendations for the duration of food deprivation for each species as this will depend upon the normal feeding frequency, diet and digestive tract transit time. It is unlikely that in most cases more than 1–2 days, food deprivation is necessary to void the digestive tract of food and digesta, with the longer time being more appropriate for species held at lower temperatures where transit time may be slower (Boucaud-Camou and Boucher-Rodoni, 1983; Mangold and Bidder, 1989). It is essential that the duration of any food deprivation prior to transport is scientifically justified and is minimized to avoid compromising health (see below) during what is likely to be a stressful event.

### Surgery and anesthesia

In humans, deprivation of food prior to anesthesia and surgery is justified because of the risk of aspiration of vomit during induction, before airway intubation and this is also normal practice prior to veterinary or experimental surgery in larger mammals. The justification for routinely depriving cephalopods of food prior to anesthesia and surgery is not established and obviously inspiration of any regurgitated digestive tract contents into the mantle does not pose the same risk to a cephalopod as aspiration of vomit in a mammal. However, there is an argument for food deprivation if the surgical procedure involves the digestive tract itself. A full stomach (e.g., *L. vulgaris*) or crop (e.g., *O. vulgaris*) may increase the risk of accidental damage by obscuring other structures (e.g., crop branch of the dorsal aorta) or limiting the plane of dissection, so food deprivation may also be justified although this would only need to be omission of one meal or about 24 h (Fiorito et al., 2015).

### Metabolic studies

Laboratory investigation of metabolism can require animals to be deprived of food or may require a reduction in the amount of food provided at each meal. The question arises at what point food deprivation itself, as part of a research study, falls within the definition of a *procedure* which would be regulated under Directive 2010/63/EU and hence should be included in protocols submitted as part of a project application to the National Competent Authority.

Under Directive 2010/63/EU the threshold for regulation is “any use of an animal covered by the Directive for experimental or other scientific or educational purposes, which may cause the animal pain, suffering, distress or lasting harm equivalent to or higher than that caused by the introduction of a needle in accordance with good veterinary practice.” It is clearly not easy to translate this definition into the period for which a cephalopod of any particular species (for example, a highly active *L. vulgaris* vs. relatively slow moving *N. pompilius*) or life stage (e.g., *O. vulgaris* paralarvae vs. egg bearing female) can be deprived of food before it exceeds the threshold for regulation. Periods of food deprivation of 24–48 h have been used prior to investigating the effect of handling on attack latency (≈24 h in *O. vulgaris*; Agnisola et al., 1996), before a feeding experiment (48 h in *E. tasmanica*; Moltschaniwskyj and Johnston, 2006) or prior to a study of anesthesia (24 h in *S. officinalis*; Gonçalves et al., 2012) based largely upon measures of oro-anal transit time for the species under study and also taking into account normal feeding frequency for the species (see above). In *N. pompilius* a period of 5 days food deprivation was used to stimulate appetite prior to feeding barium sulfate labeled shrimp to measure digestive tract transit (Westermann et al., 2002).

A number of studies have investigated the effects of various periods of food deprivation in *S. officinalis, Loligo forbesi*, and *O. vulgaris* on metabolism and these are used to make proposals regarding the regulatory threshold and also humane end points (see below).

The physiological response to food deprivation in both vertebrates and invertebrates has three phases (Lamarre et al., 2016): *Phase I* - basal metabolism is sustained following a few days food derivation utilizing dietary constituents; *Phase II* - with continued food deprivation mobilization of stored lipids occurs and for cephalopods these are primarily located in the digestive gland. A recent study shows that their role in enabling cephalopods to survive prolonged food deprivation may have been underestimated (see Speers-Roesch et al., 2016); *Phase III* - characterized by protein catabolism, it is considered to be the real indication of starvation as opposed to food deprivation Phases I and II.

Juvenile and adult animals are most likely to be used for research studies requiring food deprivation and for these life stages we propose the following regarding the threshold for regulation; periods of food deprivation that do not exceed the metabolic effects of Phase I should be considered to be below the threshold for regulation as defined by Directive 2010/63/EU (European Parliament and Council of the European Union, 2010) whilst procedures that induce the metabolic changes characteristic of Phases II and III fall within the threshold for regulation and would fall into the mild and moderate prospective severity classes respectively (European Commission, 2013). The work by Lamarre et al. (2012) and Speers-Roesch et al. (2016) did not report any mortality in the prolonged food deprivation studies. However, in a group of *O. vulgaris* (body weight 1618.3 ± 175.5 g) deprived of food there was an overall 10% mortality with individuals dying by an undetermined cause at 16, 20, and 23 days (Garcia-Garrido et al., 2010). The overall weight loss in the animals that survived the entire 27 days food deprivation was 35%. The possibility that death may occur in a food deprivation study would be likely to make the severity grading severe and such studies would require detailed justification of the scientific or other benefits vs. the harms to the animal.

The behavior of animals following prolonged food deprivation is not described in detail but in both O. *vulgaris* (Wells et al., 1983) and *S. officinalis* (Lamarre et al., 2016) reduced levels of activity are reported and in the latter there are also problems with buoyancy.

Data from the above metabolic studies can provide a guide to the durations of food deprivation exceeding the threshold for regulation under Directive 2010/63/EU and their severity classification. Table 1 proposes how the physiological consequences of food deprivation could be linked to severity classification. Research will be needed to match the relatively well-defined metabolic changes to behavioral (or other) objective indices of “pain, suffering, distress, and lasting harm.”

**Table 1:** The prospective severity classification of experimental procedures as defined in Directive 2010/63/EU together with proposals for how this could relate to periods of food deprivation in an adult *O. vulgaris* or *S. officinalis* in good health at the beginning of the deprivation.

	**Below threshold**	**Mild**	**Moderate**	**Severe**
Definition of severity from EU 2010^*^ and EC 2013^*^	Does not reach the regulatory threshold of causing pain, suffering, distress or lasting harm equivalent to, or higher than that caused by the insertion of a hypodermic needle in accordance with good veterinary practice (EU 2010)	Likely to cause the animals to experience short- term mild pain, suffering or distress as well as procedures with no significant impairment of the well-being or general condition of the animals (EC 2013)	Likely to cause the animals to experience short- term moderate pain, suffering or distress as well as procedures that are likely to cause moderate impairment of the well-being or general condition of the animals (EC 2013)	Likely to cause the animals to experience severe pain, suffering or distress or long-lasting moderate pain, suffering or distress as well as procedures that are likely to cause severe impairment of the well-being or general condition of the animals (EC 2013)
Duration of food deprivation	0–48 h (0–2d)^**^	48–120 h (2–5 d)^+^	120–288 h (5–12 d)^+^	>288 h (>12 d)
Anticipated body weight loss (%) (based on Garcia-Garrido et al., 2010)	0 ≤ 5%	<10%	~10–15%	>15%
Anticipated digestive gland weight loss (%) (based on Garcia-Garrido et al., 2010)	0–30%	30–50%	50–60%	>60%
Metabolic phase	Early Phase I	Late Phase I-early Phase II (Speers-Roesch et al., 2016, “middle phase”)	Late Phase II-Early Phase III (Speers-Roesch et al., 2016, “middle phase”–“final phase”)	Phase III- metabolic collapse (Speers-Roesch et al., 2016, “final phase”)
Metabolic status (Based on Speers-Roesch et al., 2016; Table 1)	Normal metabolism from mixed biochemical sources but by 2–3d signs of a shift from anabolic to catabolic metabolism will emerge	Progressive utilization of lipids from the digestive gland and clear shift from anabolic to catabolic metabolism in the digestive gland.	Lipid dominant metabolism supplemented by tissue protein catabolism. No glucose utilization.	Dependence on amino acid metabolism with no glucose use and depleted lipid stores
Anticipated behavioral and other possible effects	No negative effects anticipated but possible increase in food seeking behavior; experience of hunger sensation?	Progressive reduction in activity. Adaptive changes in the digestive tract such as upregulation of epithelial transport mechanisms.	Progressive reduction in activity. Suppression of digestive tract motility.	Overall decline in health; increased susceptibility to infection and skin lesions); potentially irreversible damage to critical tissues (e.g., gills). Death with prolonged deprivation^#^.

*The exact boundaries may change depending on temperature and life stage (see text for details). Based largely on studies of O. vulgaris (Garcia-Garrido et al., 2010) and S.officinalis (Speers-Roesch et al., 2016) we also indicate the underlying biochemical changes. The behavioural or other externally visible consequences of food deprivation are not well defined as indicated by the paucity of specific information in the last row (see text for details). Note that this table illustrates the principles and National Competent Authorities may have different views on severity and the above proposed classification requires validation*.

* *“EU 2010” and “EC 2013” are abbreviations of European Parliament and Council of the European Union (2010) and European Commission (2013), respectively*.

** *For comparison in an adult salmonid 48 food deprivation would be considered to be below the regulatory threshold but it is also noted that as in cephalopods there is considerable inter-species variation (Hawkins et al., 2011)*.

+ *For comparison in adult rats, food deprivation of <24 h would be classified as mild and deprivation for 48 h as moderate (European Parliament and Council of the European Union, 2010)*.

# *In O. vulgaris Garcia-Garrido et al. (2010) reported death at 16, 20, and 23 days of food deprivation*.

If food deprivation is required for whatever reason, a clear justification should be made both in the application to the National Competent Authority (if the work is covered by Directive 2010/63/EU) and reiterated in any publication. The period of food deprivation should be kept to the minimum compatible with achieving the stated objectives and the impact on all aspects of the animals' health should be carefully assessed (see ARRIVE reporting guidelines; https://www.nc3rs.org.uk/sites/default/files/documents/Guidelines/NC3Rs%20ARRIVE%20Guidelines%202013.pdf). In the case of prolonged food deprivation (e.g., Lamarre et al., 2012), the fate of the animal at the end of the period of deprivation will need to be considered and specified in the application to the National Competent Authority (NCA) in research performed under Directive 2010/63/EU (European Parliament and Council of the European Union, 2010). Whilst it is possible that after an extended period of food deprivation the animals may regain body weight when sufficient food is available, the welfare of the animal during this recovery period must be carefully assessed and there should be some assurance that the period of deprivation did not have irreversible effects such as structural protein or lipid loss in critical tissues (brain, heart, and gills) that may cause persistent suffering to the animal.

## The digestive tract as a route for substance administration

Drug administration by gavage has been used in squid (Berk et al., 2009) and could be used in cuttlefish and octopus although we are not aware of any publications. If gavage is used, care must be taken to taken to avoid damage to the brain as the gavage tube passes from the beak into the esophagus and then into the crop or stomach depending on species. The tube to be utilized should be semi-rigid and round ended, to prevent mucosal damage; measurements should be made on cadavers to estimate the length to which the tube should be inserted unless this is done using ultrasound assisted *in vivo* imaging.

Food deprivation is probably advisable to facilitate rapid absorption of any drug and prevent drug/food interaction, but the volume of substance administered should be well within the normal volumes found in the crop or stomach to minimize chances of damage or activation of visceral nociceptors (should they exist in cephalopods). Careful consideration should be given to the drug vehicle to avoid substances likely to damage the mucosa or which themselves have effects on mucosal functions (e.g., changes in pH that will alter the pH optimum of digestive enzymes and mucolytics will impair epithelial barrier function).

An alternative to gavage is to include the drug in the food. This route was used for the administration of titanium dioxide nanoparticles to *O. vulgaris* by feeding the animals on mussels that had been exposed to the nanoparticles (Grimaldi et al., 2013). Inclusion of drugs in the food (e.g., injected into a crab, a mussel, a piece of fish or incorporated into a piece of artificial diet just prior to feeding) provides a non-invasive approach to administration but there are several issues to be considered if a study using this approach is to have a meaningful outcome: (1) Although the total dose of drug in the food is known it may be difficult to know the precise amount ingested by the animal unless there is assurance that no drug has leaked into the water during food capture or ingestion and that the animal has eaten all the food. Microencapsulation of the drug will reduce leakage compared to injecting the drug into the food in solution or mixing as a powder but assessing the ingested does remains problematic; (2) The secretions of the salivary glands injected into the prey during capture and prior to ingestion contains powerful enzymes and a wide array of other bioactive substances (Cornet et al., 2014; Mancuso et al., 2014) so some consideration should be given to whether the drug is likely to be affected by the salivary secretions and rendered ineffective. Degradation of the drug by the salivary secretions could lead to false negative conclusion about drug efficacy in cephalopods; (3) As the food with the drug is likely to come into contact with the suckers, particularly in the peri–oral region, there is a possibility that the animal may reject the food based upon “taste” although micro-encapsulation could be used to prevent direct contact.

In cuttlefish, Darmaillacq et al. (2004) showed induction of a learned aversion to food (crabs) painted with quinine, which is bitter tasting to humans, although we do not know what sensation (if any) it may evoke in cephalopods. If the experimental drug evokes rejection and a learned aversion to that food in a similar manner to quinine (many alkaloids are bitter tasting and are also drugs, e.g., atropine and scopolamine), then it may not be ingested and may induce an aversion to that food if encountered again. A learned aversion leading to avoidance of the food on a subsequent occasion can also be induced if the ingested food causes the animal to be ill. In humans the analogous situation would be a food that induced nausea and vomiting (Andrews and Sanger, 2014).

As it may not be possible to predict if a drug will have an adverse effect on the animal it should not be given in the food most commonly used, otherwise if the animal does develop an aversion maintenance of the normal feeding regime may not be possible.

## Research on digestive physiology and diets for cephalopods: impact of directive 2010/63/EU and other regulations and policies

Directive 2010/63/EU and related mandated minimal recommendations on the accommodation and care of animals, include species specific sections that are relatively detailed for vertebrates, but no equivalent indications are provided for cephalopods. However, the “general section” of the Directive (European Parliament and Council of the European Union, 2010) applies to all species and includes some general principles of feeding (Annex III, 3.4) which we outline here as they are relevant to the subsequent discussion of cephalopod diets.

The key elements included under the heading “Feeding” in the Directive are:

- Form, content and presentation of the diet should meet the nutritional and behavioral needs of the animal;
- Diet should be palatable and non-contaminated and precautions taken to minimize chemical, physical and microbiological contamination;
- Individual animals should be able to access food, with sufficient space provided to limit competition.

These principles embodied in the Directive and national transposition legislation, are reflected in the Guidelines on the Care and Welfare of Cephalopods (Fiorito et al., 2015), and also included in a simplified version, in the Code of Practice on Housing and Care published by the United Kingdom Home Office (see section on Cephalopods; https://www.gov.uk/government/publications/code-of-practice-for-the-housing-and-care-of-animals-bred-supplied-or-used-for-scientific-purposes).

Directive 2010/63/EU applies to the protection of animals used for scientific purposes but as many aspects of this review also apply to animals in an aquaculture setting we draw attention to the following regulations: Council Directive 98/58/EC on the protection of animals kept for farming purposes (Council of the European Union, 1998); Regulation (EC) N° 882/2004 on official controls performed to ensure the verification of compliance with feed and food law, animal health and animal welfare rules (European Parliament and Council of the European Union, 2004); and Council Regulation (EC) N° 1099/2009 on the protection of animals at the time of killing (Council of the European Union, 2009).

The identification of optimal dietary formulations (natural or synthetic) for use in cephalopods in the laboratory and aquaculture, at all life stages, requires research into their effects on growth and metabolism (Sykes et al., 2006; Navarro et al., 2014). There is debate about whether research into different diets falls within the remit of Directive 2010/63/EU (see Sykes et al., 2012) so the issues of concern are discussed here. Firstly, investigation of the effects of different diets is clearly “research” as the outcome is not known and an experimental approach is required to obtain the answer so does not come under the exempt category of “non-experimental agricultural practices” (Article 1, 5a). Secondly, the response of the animal to a novel experimental diet may not be immediately apparent so there is a potential that the animal may experience suffering, distress or lasting harm that exceeds the threshold for regulation. Until the novel diet is studied its effects on the animal cannot be fully assessed. Third, many studies are directed at identification of diets or environmental factors to improve survival of paralarvae in species such as *O. vulgaris* where mortality in aquaculture conditions from hatchling to the benthic phase is ≈100% in most of the studies and has changed little over 20 years (Villanueva, 1995). Note that the survival rate in the wild is not known so comparison with survival rates in laboratory condition is not possible. In studies of diet and environmental changes, mortality/survival rates are often used as one of the outcome measures. The aim of many paralarvae studies is to modify the diet, light or temperature to improve survival rates so mortality is a valid outcome measure. However, the severity classification of procedures would classify as “severe” toxicity testing where death is an end point or where there is a severe impairment of well-being or general condition (e.g., see paper by Feyjoo et al., 2011). The effect of a novel diet on survival cannot be predicted, so it is possible that the mortality may be either higher or lower than a current feeding regime. Whilst the degree of suffering which may be experienced by a paralarvae before death is not known, this discussion illustrates the regulatory challenges raised by the inclusion in Directive 2010/63/EU of cephalopods from the time of hatching.

## The potential impact of experimental procedures on the cephalopod digestive tract: issues to consider

The potential impact of any experimental procedure on functionality of the digestive tract should be assessed as part of experimental planning. For investigators whose research is regulated by Directive 2010/63/EU (see below) a full assessment of the effects of an experimental investigation and assessment of the anticipated harms (to the animal) and potential benefits (of the answer to the research question) is an essential component of the application for authorization to the National Competent Authority. There have not been any formal studies on the effect of common procedures on the cephalopod digestive tract but below we comment on the most likely areas of concern to stimulate discussion and raise awareness of the issues. As for other issues discussed in this is overview the specific effects on the digestive tract of any intervention are likely to be affected by life stage and species.

### Anesthesia and surgery

A number of authors have commented that cephalopods recover rapidly from brief periods of general anesthesia, not usually involving surgery, with reports of food ingestion within 10–15 min of recovery anesthesia (Joll, 1977; Gonçalves et al., 2012). Food ingestion indicates an unimpaired attack response but does not necessarily imply that the digestive tract itself is unaffected by anesthesia, although it is suggestive. Whilst an anaesthetic may have an effect on the digestive tract by acting on the brain regions regulating the extrinsic innervation (visceral and sympathetic nerves; Young, 1967, 1971) a direct action on the digestive tract enteric nerves and muscle is also possible. For example, magnesium chloride is commonly used as an anesthetic agent in cephalopods (Fiorito et al., 2015) and in the isolated stomach and rectum of *Loligo pealii* it has an inhibitory effect on contractions and tone (Bacq, 1934). This inhibitory effect on the digestive tract is also consistent with the bradycardia and reduced stroke volume produced by magnesium chloride on the isolated systemic heart of *O. vulgaris* (Pugliese et al., 2016). For both the heart and digestive tract, the most likely mechanism by which magnesium chloride has its inhibitory effects is by interfering with calcium fluxes, but this requires confirmation by experiment. If digestive tract motility is inhibited for the duration of anesthesia it is likely that transit time will be prolonged until motility is normalized.

Surgery of the digestive tract and in particular lesions affecting the innervation (e.g., sympathetic nerves, gastric ganglia) will not surprisingly affect digestive tract function (e.g., Best and Wells, 1983) and hence impact the overall welfare of the animal. The impact of surgery outside the digestive tract on digestive tract functionality is not known, but if nociceptors are activated by the surgery (and analgesics are not provided) then neural and endocrine pathways regulating digestive tract function may be affected. Particular care needs to be taken to consider the likely effects of brain lesions on the ability of the animal to recognize, capture and ingest food and hence maintain normal metabolism. For example, *O. vulgaris* with lesioned anterior and posterior basal lobes are unable to feed themselves (Wells, 1978) so if there is a need to study these animals for more than a few days consideration will need to be given to how to ensure adequate nutrition to maintain health.

Currently, no analgesics have been used as part of the anesthesia protocol in cephalopods (Fiorito et al., 2015) but if potential substances are identified their pharmacological effects on the digestive tract should be ascertained. For example, opioid receptor agonists (e.g., morphine and synthetic derivatives) are used as an analgesic in mammals and are associated with constipation as a side effect, as amongst other actions they modulate transmission in the enteric nervous system to reduce transit (De Giorgio et al., 2007). Delta-opioid receptors have been identified in the digestive tract (Sha et al., 2012) and kappa-opioid receptors in *O. vulgaris* (Zarrella et al., 2015) but functional effects of their activation have not been investigated.

### Nociception and stress

In mammals, activation of somatic and visceral nociceptors inhibits gastric and intestinal motility contributing to an overall increase in oro-anal transit time via reflex and endocrine mechanisms. The nociceptors activate spinal and supra-spinal pathways modulating the sympathetic outflow to the digestive tract and the secretion of adrenaline from the adrenal medulla (Janig, 2013; Janig and Mclachlan, 2013).

Although mechano-nociceptors have been described in cephalopods (Crook et al., 2011, 2013, 2014; Alupay et al., 2014) in locations equivalent to somatic nociceptors in vertebrates their central projections are not known so we are unable to speculate if information from these nociceptors can influence the innervation to the digestive tract (visceral nerves and “sympathetic” nerves; Young, 1967). The existence of visceral nociceptors has not been investigated in cephalopods but there is structural and behavioral evidence for the existence of visceral afferents (Young, 1960, 1967). Nothing is known about the responses of the cephalopod digestive tract or the cardiovascular system (also modulated by the visceral nerves) to noxious stimuli but it would be surprising if neither system was affected. Non-painful, but unpleasant stressful stimuli such as those produced by restraint or hypoxia are also likely to affect digestive tract function via the innervation or secretion of “stress” hormones. For example, removal of *E. cirrhosa* from water for 5 min results in elevation of haemolymph dopamine levels (Malham et al., 2002), which could act on the crop where *in vitro* studies in *O. vulgaris* have shown dopamine to increase tone (Andrews and Tansey, 1983b) or alternatively the neurones in the gastric ganglion could be the target as there is histochemical evidence for its presence (Juorio, 1971) and hence dopamine receptors are likely to be present.

Both noxious and non-noxious but stressful external stimuli may also have both acute and chronic effects on the digestive tract via up or down regulation of genes in critical control locations such as the gastric ganglion. A diverse range of genes has been implicated in the response of octopus to environmental stressors (reviewed in Di Cosmo and Polese, 2016) but the biological effects of the gene products on the physiology of the digestive tract and its control requires investigation.

### Drug administration

A drug given systemically and being used as part of a research project to investigate one system (e.g., brain neurochemistry and effects on behavior) may also have unintended effects on control of food intake or the functioning of the digestive tract. For example, catecholamines are present in the brain (Tansey, 1980) and in neurones in the digestive tract and cardiovascular system of cephalopods (Andrews and Tansey, 1983a; Versen et al., 1999). Drugs used to investigate the role of catecholamines in brain functioning (e.g., learning and memory) either by depleting nor-adrenaline (e.g., reserpine; Tansey, 1980) or acting as competitive antagonists of adrenergic receptors (e.g., phentolamine; Versen et al., 1999) could affect digestive tract motility as nor-adrenaline stimulates contractile activity in the crop, stomach and intestine of *O. vulgaris* (Andrews and Tansey, 1983b). The potential problems are further illustrated by scopolamine (a non-selective muscarinic acetylcholine receptor antagonist) used to investigate memory recall in *O. vulgaris* (Fiorito et al., 1998) but as acetylcholine (receptor not characterized) has inhibitory effects on digestive tract motility (Andrews and Tansey, 1983b) this effect may be lost in animals treated with scopolamine, allowing excitatory effects to predominate. In neither paper using reserpine (Tansey, 1980) or scopolamine (Fiorito et al., 1998) were overt effects on the digestive tract reported but neither were they investigated explicitly and such effects may be subtle.

### Developmental changes

The publication of the genome of *O. bimaculoides* (Albertin et al., 2015) will be a major stimulus to cephalopod research. The intended and potential unintended effects of gene expression modifications (e.g., gene knock out, gene over expression) will need to be considered during project design and plans put in place to monitor adverse (or even beneficial) effects. Of particular concern would be modification to genes which may affect the development of the digestive tract including the neural control mechanisms. Exposure of recently spawned eggs to abnormal environmental condition can also produce developmental changes as demonstrated by an increase in malformations, early hatching and mortality in squid eggs exposed to sea water at 2°C above ambient (Rosa et al., 2012) or eggs of *O. vulgaris* exposed to an increase of 3°C (Repolho et al., 2014).

## Reduced food intake as a humane end-point in procedures: what are the limits?

A humane end point is the earliest point at which a specific intervention must be made to end an animal's suffering and for each procedure under Directive 20110/63/EU. Humane end-points are specified in the application to the National Competent Authority and describe the type of suffering that may occur, its magnitude and duration and these judgements contribute to the “prospective assessment of severity” of a procedure which is also indicated in application to the NCA (see below).

Typical interventions as the humane end point is reached could include: (i) removing the animal from the study; (ii) providing analgesia to alleviate pain or by treating other symptoms unrelated to the experimental outcomes but this is reliant on validated treatment being available and knowledge that they will not affect the primary experimental outcomes; (iii) humanely killing the animal and/or terminating the study.

There are three different types of study where the consequences and limits of reduced food intake need to be considered and these are discussed below to illustrate the issues. For completeness, we also include the special case (see below) of research involving senescent cephalopods.

### Investigation of the effects of food deprivation

There are a number of studies aimed at investigating the effects of different periods of food deprivation on a cephalopod (e.g., studies of *O. vulgaris*, Garcia-Garrido et al., 2010; *S. officinalis* studies of Lamarre et al., 2012, 2016; Speers-Roesch et al., 2016). In such studies the duration of food deprivation is pre-determined and justified in terms of the anticipated harms to the animals vs. the potential scientific (or other) benefits of the study. Weight loss is an expected outcome of these studies, but if the protocol in the NCA submission has set a limit for the degree of weight loss (e.g., 20% over 14 days in adult *O. vulgaris*) then if this is exceeded, the animal would be removed from the study immediately. Humane end-points indicating that the food deprivation is having additional deleterious effects could include loss of the ability to maintain normal position in the water column, autophagy, skin lesions or an excessive reduction in the size of the digestive gland (e.g., measured by ultrasound; for a description of welfare monitoring parameters see Table 5 in Fiorito et al., 2015). If these signs were used as humane end-points then, if observed, that animal would be removed from the study.

### Interventions intended to modify food intake

There are a number of studies in which an intervention (e.g., an agonist with potential anorexic effects, administration of a pathogen or a nerve lesion such as gastric ganglion ablation) is intended to, or is likely to, modify food intake. In this type of study the issue is what limits should be placed on the reduced food intake? For example, for how many days should an animal be allowed to go without food before intervention? The duration will be related to the scientific outcomes; if the aim is to show that an intervention (e.g., investigating modulation of food intake by members of FMRFamide family; Walker et al., 2009) affects food intake then a period of 24 h may be sufficient to show an acute effect, but if there is a need to know the duration of any effect then longer may be needed. The duration of the study will be the minimum compatible with the scientific objectives assuming that the duration can be justified in the project application. Decisions about how long an animal should go without food or have reduced food intake before intervention should take into account metabolic studies (e.g., Garcia-Garrido et al., 2010; Lamarre et al., 2012, 2016; Speers-Roesch et al., 2016) but until more detailed information is available about the effects of food intake on physiology and behavior of the animals we would advise adoption of the precautionary principle in studies where food intake is likely to be affected.

### Procedures affecting food intake as an unintended outcome

In some cases experiments include a procedure that may affect food intake although this is not either the primary intent of the lesion or topic of study. In this case the reduced food intake is viewed as an “adverse” or “side” effect of the procedure. This type of study differs from food deprivation described above in that the effect on food intake is unintended rather than being a primary objective of the study but in both cases the issues relating to the duration of reduced food intake are the same.

### Senescence

Markedly reduced or absent food intake is a well-known feature of senescent cephalopods occurring in post-mating adults and in females during egg-brooding (Anderson et al., 2002). Although this is a normal terminal life-stage in the wild, if the animal is being kept in the laboratory to study the physiology of senescence (e.g., how is appetitive drive inhibited?) then as the animal is likely to experience some suffering it arguably comes within the scope of Directive 2010/63/EU (see Smith et al., 2013, for discussion). As the reduction in food intake progresses the animals will rapidly pass through metabolic phases I, II described above in animals deprived of food and will probably spend the longest time in phase III until death ensues (Moltschaniwskyj and Carter, 2013). Studying senescence is further complicated because animals not only stop eating but may develop other symptoms such as cataracts, skin lesions and increased, but uncoordinated, locomotor activity (Anderson et al., 2002; Sykes et al., 2012; Sykes and Gestal, 2014) none of which can be alleviated. If studies of the digestive changes accompanying reproduction and senescence are to be investigated a critical question will be whether it is necessary to study the animal until death to answer the scientific question posed? We would argue that understanding the mechanism of food intake suppression only requires a relatively short period of study (e.g., a week) but if the topic of interest is whether the enteric nervous system shows signs of degeneration with age, as occurs in mammals (Hetz et al., 2014; Saffrey, 2014), then longer periods of study may be required.

## Recommendations and research questions to enhance cephalopod health and welfare

This review has drawn attention to the numerous close relationships between the normal physiological functioning of the digestive tract in cephalopods and their health and welfare. Although the relationships between normal digestive tract function, health and welfare may appear obvious, conforming with regulatory requirements (particularly Directive 2010/63/EU) necessitates developing a more evidence based approach to ensuring adequate nutrition and identification of biomarkers of health and welfare.

The text below summarizes some of the key points and areas which we consider require research.

- Reporting feeding regimes in publications is variable so a consistent approach should be adopted to include as a minimum: type of food; amount of food (weight or as a % body weight); feeding frequency and time. This will facilitate meta-analysis of diets.
- Cephalopods for research are frequently taken from the wild and may have been exposed to ingested toxins (e.g., domoic acid) and parasites (e.g., Aggregata); these are considered as potential confounding factors in research which may be unidentified prior to allocation to experimental groups. The impact of parasites and toxins on health and welfare requires research.
- Techniques for non-invasive investigation of digestive tract physiology are required to enhance routine health and welfare assessment (for review see Ponte et al., 2017), to improve understanding of the effects of experimental interventions and also to develop humane end-point criteria for regulated procedures.
- Little is known of relationships between behavior and digestive tract functionality in cephalopods. For example: would digestive tract obstruction result in behavioral change due to visceral nociceptor activation? What behavioral changes accompany food deprivation of different durations?
- Periods of food deprivation may be required for various types of research and we considered this in relation to knowledge of cephalopod metabolism and the regulatory threshold and prospective severity assessment; deprivation >2d is proposed to reach the threshold for regulation under Directive 2010/63/EU in the “mild” category and >12d would be “severe.”
- The potential negative impact of experimental interventions such as food deprivation, anesthesia (e.g., inhibition of smooth muscle contraction by magnesium chloride) and surgery, (e.g., brain lesion), pain (e.g., activation of nociceptors) and stress (e.g., weighing in air), drug administration (e.g., cholinergic and adrenergic receptor antagonist impacting digestive tract innervation) and genetic modification on the functioning of the digestive tract are considered from the perspective of animal welfare.
- Research on the digestive tract physiology is needed to understand constraints of *O. vulgaris* aquaculture and in particular the maturation of function and control mechanisms (e.g., gastric ganglion) and changes occurring between the paralarvae and settlement phases where dietary changes occur and there are likely to be changes in the digestive tract microbiome.
- Improved understanding of all aspects of the physiology of the digestive tract (food intake regulation, motility, secretion, absorption, neural, and endocrine control) in a diverse range of species will play a key role in ensuring optimal health and welfare of cephalopods in the laboratory, aquaculture and display aquaria.

Good animal welfare and good science are inextricably linked (see Hubrecht, 2014, for review). We hope that this paper will stimulate research on the digestive system which in addition to providing novel insights into the physiology will also enhance the welfare of cephalopods in both research and aquaculture.

## Author contributions

AVS and EA are equal first authors. All authors contributed to the manuscript and drafted, edited and approved the final version of the manuscript.

### Conflict of interest statement

The authors declare that the research was conducted in the absence of any commercial or financial relationships that could be construed as a potential conflict of interest.
